# Cytoskeletal Dynamics in Epithelial-Mesenchymal Transition: Insights into Therapeutic Targets for Cancer Metastasis

**DOI:** 10.3390/cancers13081882

**Published:** 2021-04-14

**Authors:** Arpita Datta, Shuo Deng, Vennila Gopal, Kenneth Chun-Hong Yap, Clarissa Esmeralda Halim, Mun Leng Lye, Mei Shan Ong, Tuan Zea Tan, Gautam Sethi, Shing Chuan Hooi, Alan Prem Kumar, Celestial T. Yap

**Affiliations:** 1 Department of Physiology, Yong Loo Lin School of Medicine, National University of Singapore, Singapore 117593, Singapore; phsarpi@nus.edu.sg (A.D.); phsdes@nus.edu.sg (S.D.); e0512812@u.nus.edu (V.G.); e0176787@u.nus.edu (K.C.-H.Y.); phsceh@nus.edu.sg (C.E.H.); e0370773@u.nus.edu (M.L.L.); e0013225@u.nus.edu (M.S.O.); phshsc@nus.edu.sg (S.C.H.); 2 Department of Pharmacology, Yong Loo Lin School of Medicine, National University of Singapore, Singapore 117593, Singapore; phcgs@nus.edu.sg; 3 Cancer Science Institute of Singapore, National University of Singapore, Singapore 117593, Singapore; csittz@nus.edu.sg; 4 Cancer Translational Research Programme, Yong Loo Lin School of Medicine, National University of Singapore, Singapore 117593, Singapore; 5 National University Cancer Institute, National University Health System, Singapore 119074, Singapore

**Keywords:** actin cytoskeleton, epithelial to mesenchymal transition, metastasis, multidrug resistance

## Abstract

**Simple Summary:**

The epithelial to mesenchymal transition (EMT) is a well-documented process in the study of cancer metastases. The cytoskeleton is an intricate network involved in various cellular activities and impacts cell shape, division, trafficking, and motility. However, several functions and activities of the cytoskeleton, which plays a pivotal role in EMT, are not fully understood. This review aims to provide significant insights into the cytoskeleton’s physiological functions and the crucial role in the EMT process. Our review focuses on the participation of actin filaments, intermediate filaments, and microtubules in promoting EMT and their influence on cancer metastasis. We have also highlighted potential therapeutic targets associated with EMT activation for clinical intervention. A better understanding of multi-drug resistance (MDR) mechanisms in cancer cells with the cytoskeleton could accelerate the discovery of new therapies for aggressive cancer.

**Abstract:**

In cancer cells, a vital cellular process during metastasis is the transformation of epithelial cells towards motile mesenchymal cells called the epithelial to mesenchymal transition (EMT). The cytoskeleton is an active network of three intracellular filaments: actin cytoskeleton, microtubules, and intermediate filaments. These filaments play a central role in the structural design and cell behavior and are necessary for EMT. During EMT, epithelial cells undergo a cellular transformation as manifested by cell elongation, migration, and invasion, coordinated by actin cytoskeleton reorganization. The actin cytoskeleton is an extremely dynamic structure, controlled by a balance of assembly and disassembly of actin filaments. Actin-binding proteins regulate the process of actin polymerization and depolymerization. Microtubule reorganization also plays an important role in cell migration and polarization. Intermediate filaments are rearranged, switching to a vimentin-rich network, and this protein is used as a marker for a mesenchymal cell. Hence, targeting EMT by regulating the activities of their key components may be a potential solution to metastasis. This review summarizes the research done on the physiological functions of the cytoskeleton, its role in the EMT process, and its effect on multidrug-resistant (MDR) cancer cells—highlight some future perspectives in cancer therapy by targeting cytoskeleton.

## 1. Introduction

Cancer metastases continue to be a significant clinical hurdle in cancer diagnosis and treatment. Hence, much focus has been given to the so-called epithelial-mesenchymal transition (EMT), a vital process by which epithelial cells change biochemically to achieve mesenchymal phenotypes and is a critical part of cancer metastases. EMT can be classified into three sub-processes. The first comprises EMT’s role during developmental processes, such as gastrulation [1,2,3,4,5]. The second sub-process of EMT is involved in the wound repair and fibrosis processes, which are activated by inflammation [1]. The third sub-process of EMT occurs during the cancer progression, which aids the invasion and metastasis of tumor cells to distant sites and promotes the chemoresistance capabilities of these cells [1,6,7,8]. This review paper primarily focuses on the third sub-process of EMT.

Polarized, non-motile epithelial cancer cells undergo EMT, wherein they lose cell-cell adherence through the loss of cell junctions, such as the adherens and tight junctions [9,10]. The apical-basal polarity of the epithelial cells also changes to a front-rear polarity, with cytoskeletal reorganization, which induces changes in cell shape and a restructuring of the cells’ attachment to the extracellular matrix (ECM) [10]. Once EMT is completed, the cells become mesenchymal and motile. Underlying these phenomena are the molecular changes occurring in the cells. TGF-β, IGF-II, FGF, and EGF signaling promote EMT by activating transcription factors such as Snail, Twist, and ZEB, and they mediate gene expression changes in the cells [10,11,12]. Epithelial cell markers such as E-cadherin, a significant component of the cell junctions, are downregulated [9,10]. On the other hand, N-cadherin, fibronectin, vitronectin, and MMPs are transcriptionally upregulated by these three transcription factors, contributing to the mesenchymal phenotypes of the cells [4,10,13,14]. Originally, EMT was defined as a morphological conversion, but recent advances in biochemical studies have revealed that EMT acts as a central mechanism for carcinoma progression and metastasis. The transcriptional program controlling trans-differentiation and morphological changes during EMT has been comprehensively studied and documented. In contrast, the dynamic remodeling of the cytoskeleton and how it is regulated still lacks comprehensive understanding; especially, the structural mechanism of how the cytoskeleton is remodeled is still being deciphered. Moreover, rearrangement of the actin cytoskeleton into F-actin stress fibers during EMT also aids the formation of membrane ruffles and lamellipodia, promoting the motility of the resultant mesenchymal cells [9,10,11]. This review explores the role of the actin cytoskeleton, microtubules, and intermediate filaments in EMT and how these cytoskeleton proteins can be used as a potential biomarker.

### EMT/MET in the Metastatic Cascade

**Figure 1:**
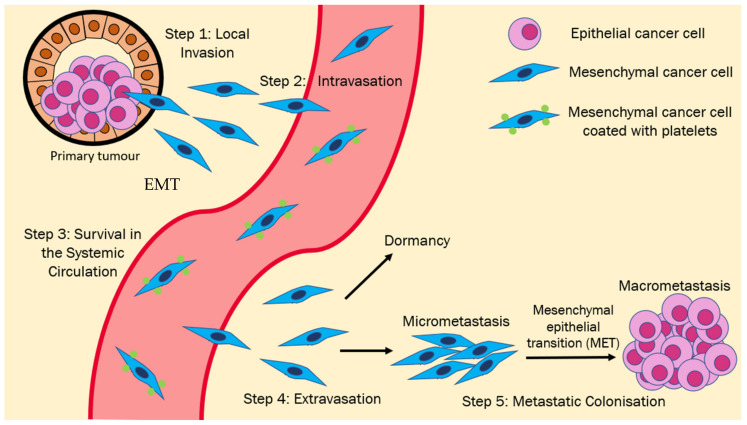
EMT-MET model for the metastatic cascade: Epithelial cancer cells undergo EMT, which causes them to lose their cell-cell junctions and gain the ability to invade the surrounding tissue parenchyma (Step 1). These EMT-induced cells may then intravasate into the systemic circulation (Step 2) and must survive in the circulation (Step 3) before reaching the target organ site. Upon reaching the target organ site, the cells must then extravasate into the tissue parenchyma (Step 4), following which they may either enter a state of dormancy or form micro metastases. Subsequent development into clinically detectable and potentially life-threatening macro metastases requires MET activation (Step 5).

EMT is a reversible process—EMT followed by a MET is crucial for cancer metastasis [15]. This cancer metastasis process is well-documented and consists of the following key steps that highlight the importance of EMT in the metastasis process (Figure 1).

EMT in Primary Tumors

The first step in cancer metastasis is the local invasion of the tissues surrounding the primary tumor [16,17]. In order to do so, the epithelial cancer cells need first to lose their cell-cell junctions and become motile. This is followed by the degradation of the underlying basement membrane and ECM [18,19,20]. The ability to degrade the ECM and invade into the surrounding tissue parenchyma is obtained by activating the EMT program, where the epithelial cancer cells transform into a more invasive and motile mesenchymal state [21,22]. During EMT, adherens junctions’ dissolution is stabilized by the cleavage and degradation of E-cadherin at the cell membrane [9]. Furthermore, the expression of E-cadherin is suppressed by core transcription factors (TFs) of the EMT program, namely Snail1, Snail2, Zeb1, and Zeb2 [23,24,25]. Apart from repressing E-cadherin expression, the EMT TFs are also responsible for downregulating the expression of other epithelial genes such as desmoplakin, plakophilin, and plakoglobin [10,26], which are crucial for the formation of desmosomes intercellular junctions that have been reported to work synergistically with adherens junctions to strengthen epithelial cell-cell contact [27]. Hence, the dissolution of these intercellular junctions during EMT allows the cancer cells to separate from each other, thus promoting migration [28].

EMT in Intravasation

Following the local invasion of the tissue parenchyma, the cancer cells must cross the endothelial barrier via an intravasation process to be disseminated in the systemic circulation. As the epithelial cells change to a mesenchymal phenotype, such a transformation promotes cell migration and the creation of actin-rich protrusions. Matrixins (MMPs) are actively involved in the invasion—studies have shown that MMP-2 and MMP-9 promote metastatic pathways, such as ECM degradation, cell proliferation, apoptosis, invasion, and morphological changes [29,30]. The increased activity of MMPs results in the enzymatic degradation of adherens junctions and ECM fibers, which increases cancer cell motility and helps break through the basement membrane and the invasion of neighboring tissue [31]. EMT also facilitates intravasation by promoting tumor angiogenesis that can promote tumor spread [32,33].

EMT in the Systemic Circulation

Following the entry into the systemic circulation, cancer cells must overcome several challenges before reaching the target organ site, the first of which is anoikis. Cancer cells lose integrin-mediated anchorage to the ECM upon entering the systemic circulation. As interactions between integrins and the ECM produce pro-survival signals, a lack of these interactions halts the production of these signals, eventually leading to the onset of anoikis, a form of programmed cell death [28].

EMT in Extravasation

Upon reaching the target organ site, the cancer cells can cross the endothelial barrier in an extravasation process to reach the parenchyma. Upon reaching the parenchyma, the tumor cells establish integrin-mediated adhesions with the ECM, enabled by the filopodium-like protrusions (FLPs), which are essential for metastasis. Experimental data has shown a direct correlation between tumor cells’ ability to create FLPs and their mesenchymal states, and the FLPs formation can be prompted by the expression of Twist1 and Snail1 [34]. In 2018, podocalyxin (PODXL), a cell surface protein whose expression is upregulated during EMT, was found to be essential for mediating the extravasation process of human breast cancer cells by interacting with actin cytoskeletal linker protein ezrin, thereby causing cytoskeletal rearrangements that promote the transition of the cancer cell into a shape that is optimal for extravasation [35].

Reversion of EMT in Colonization and Establishment of Macrometastases

After reaching the target tissue parenchyma, the disseminated tumor cells (DTCs) can either go into a state of dormancy where they stop proliferation [36] or go on to form micrometastases that may eventually develop into much larger macrometastases. As histological analyses have revealed that macrometastases exhibit epithelial phenotypes rather than mesenchymal [37], the mesenchymal-to-epithelial transition (MET) theory was proposed to explain this phenomenon. This theory posits that DTCs undergo MET to revert back from a mesenchymal state to an epithelial state that allows them to proliferate at the metastatic site and develop into macrometastases. There is some experimental evidence to support MET in cancer metastasis, albeit scarce. Tsai et al. showed in a mouse skin tumor model that the reversion of EMT at the metastatic site via the withdrawal of a Twist1-inducing signal was required for the formation of distant metastases [38]. Additionally, Ocaña et al. found that the silencing of EMT inducer Prrx in BT-549 human breast cancer cells was required for metastatic colonization of the lungs. In fact, silencing both Prrx and Twist1 simultaneously resulted in increased metastatic foci [39]. Taken together, these two studies present strong evidence for the occurrence of MET at the metastatic site.

## 2. Structure and Functions of the Cytoskeleton

The cytoskeleton is a dynamic and adaptive network of filamentous and tubular protein polymers in the cytoplasm, which provides structural support for cells. The cytoskeleton consists of three major components, which are microtubules, microfilaments, and intermediate filaments. As a fundamental structure, the cytoskeleton serves multiple roles in cells. It compartmentalizes the organelles and other cellular contents, controls the cell’s shape and movement, and enables the communication between the cell and extracellular environment. The component polymers that make up the cytoskeleton, together with their regulatory proteins, continuously reorganize themselves in response to a stimulus to support the biological processes and functions.

### 2.1. Microfilament

Microfilaments are made of actin monomers polymerized into asymmetric strands with barbed and pointed ends [40,41]. An abundance of actin-binding proteins crosslinks and rearrange the thin filaments into organized and stiff actin filament networks, such as bundled networks and branched networks. The actin cytoskeleton is a critical component in a broad diversity of cellular events, ranging from cell motility, cell differentiation, vesicular trafficking to cell proliferation and cell death regulator [42]. It affects the structure and motility of a cell [43], aids in muscle contraction [44], cytokinesis [45], and interacts with myosins to transport vesicles within the cell [46,47]. The actin cytoskeleton’s ability to participate in various cellular processes is mainly dependent on its intrinsic dynamic reorganization, which continually happens under the regulation of actin-binding proteins (ABPs) in response to cellular changes [48]. More than a hundred ABPs fall into seven prominent families, namely actin-monomer-binding proteins, severing proteins, nucleation proteins, actin filament polymerases, capping proteins crosslinking proteins, and filament-binding proteins [40].

### 2.2. Microtubule (MT)

Microtubules are composed of numerous tubulin subunits made of homologous α- or β-dimers. Similar to microfilament, the microtubule is highly dynamic [49], whereby it alternates between the states of gradual extension and rapid shortening [50]. It also has a plus end and a minus end, where β-subunits and α-subunits are exposed, respectively. Microtubule assembly is regulated by microtubule-binding proteins (MTBPs), including stabilizers, destabilizers, capping proteins, bundlers/cross-linkers, molecular motors, cytoplasmic linker proteins (CLIPs), and cytoskeletal integrators [49].

Microtubules have various physiological functions like the microfilament counterpart, where it is critical for cell cycle, intracellular trafficking, cell growth, and death. The balance between assembly and disassembly of microtubules requires tight regulation to ensure proper function being executed inside cells. For instance, during mitosis, disassembly of pre-existing MT network coupled with the assembly of new MTs to form the mitotic spindles is the pre-requisite for the cell to proceed with mitotic phases. When cells are exiting mitosis, the reverse process, where mitotic spindles resolve and MT network reforms, would result in two functional daughter cells [49,51].

### 2.3. Intermediate Filament (IF)

The intermediate filament protein is characterized by a long, rod-like, α-helical, coiled-coil structure, with both ends flanked by additional residues [52]. Unlike microfilament and microtubule, mature intermediate filaments are not polarized and lack directionality. Compared to the other two cytoskeletal components, intermediate filaments are more stable with less fracturing in terms of biochemical properties, with no know motor proteins to travel along [41]. Intermediate filaments provide the structural support for the cell, where the extensive intermediate filament network in association with plasma membrane reinforces the shape and morphology of the cell [52,53,54,55]. They also respond to external mechanical stresses to transduce the signal into cells.

### 2.4. Cytoskeleton in Cancer Progression and Metastasis

It has been long known that altered cytoskeleton is crucial for the development of many pathological conditions, including cancer. Due to the essential roles of cytoskeleton in cells, many hallmarks of cancer require the participation of at least one cytoskeletal component. For instance, in the case of the actin cytoskeleton, extensive studies have shown that the microfilaments and ABPs are crucial for resisting cell death, sustaining growth and proliferation, promoting invasion and metastasis, inducing angiogenesis, and avoiding immune response [42,56,57,58].

Perhaps one of the most established roles of the actin cytoskeleton in cancer progression is its ability to influence the metastasis of cancer cells. Numerous studies have shown that the actin cytoskeleton is reorganized at distinct parts of cells like invadopodia, enabling the movement and migration of cells [59]. Moreover, such alternation of the cytoskeleton also aids the transformation of cells from a stationed epithelial type into a migratory mesenchymal type (discussed in detail later). A large group of ABPs belonging to different types and families plays a critical role in regulating many aspects of metastasis, highlighting the importance of actin dynamics and regulation during metastasis. These family members include the Rho GTPases [60], depolymerization factors the gelsolin family members and ADF family member cofilin [61], actin motor proteins like myosin family members [62], actin nucleation and branching factors Arp2/3 and its regulators [63], and capping proteins like CapG [64]. Though less understood, there is emerging evidence showing that microtubule can play a key role in metastasis. The role of microtubule in metastasis regulation might be subtype-specific, where α-Tubulin and βIII-tubulin have been linked to metastasis [65,66].

Moreover, microtubules can regulate metastasis via the crosstalk with actin. One evidence comes from a gastric adenocarcinoma study that microtubule alternation promotes cell motility via Rho GTPase [67]. Several classes of intermediate filaments have been shown to be mediators of metastasis. Vimentin has been shown to be promoting both cell motility and EMT [68]. In fact, one of the most widely used mesenchymal markers is vimentin, highlighting the critical roles of intermediate filaments in metastasis-related changes. Utilizing the highly interactive property among the cytoskeleton components, it is worth identifying new cooperative patterns and proteins which interconnect different cytoskeletal components to aid cancer metastasis.

## 3. Role of Cellular Cytoskeleton in EMT

**Figure 2:**
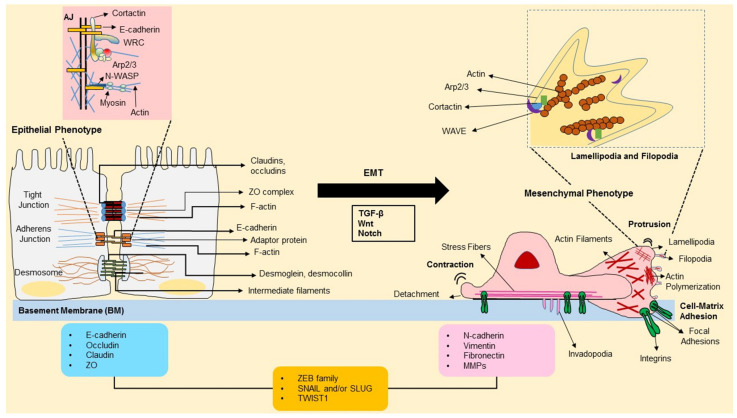
Layout of an epithelial-mesenchymal transition (EMT) transition process and forward migration of cells. Epithelial cells are bound together by tight junctions, adherens junctions, and desmosomes. The adherens junctions are cadherin-based and actin filament-associated cell-to-cell junctions that are composed of defined protein complexes. Epithelial cells are tightly secured to the basement membrane via highly specialized integrin-mediated attachment structures. Signaling pathways are said to trigger the EMT process—propagated by various EMT-TFs, such as ZEB, SNAIL, and TWIST that curb gene expression (listed in the blue box) related with the epithelial state and induce expression of genes associated with the mesenchymal state (listed in the pink box). Mesenchymal cells contain vimentin-based intermediate filaments and use integrin-containing focal adhesions to attach to the ECM. In contrast to epithelial cells, mesenchymal cell migration presents a leading and trailing edge and an extensively reorganized cytoskeleton. Lamellipodia is formed by polymerization of actin by the WAVE-Arp2/3 nucleation mechanism.

The cytoskeleton is a pivotal contributor to the cell’s structural framework and is responsible for the mechanical strength and integrity needed to establish cell shape and movement. During EMT, the epithelial cytoskeleton is restructured, such that loss of cell polarity, disruption of cell-cell junctions, and degradation of the underlying basement membrane, and reorganization of the extracellular matrix (ECM) occur (Figure 2). Then the cells become motile and acquire invasive capacity [4]. The following sections describe the essential role of the cytoskeleton in the EMT process.

### 3.1. Actin Cytoskeleton

Actin is one of the essential components of the cytoskeleton, and the remodeling of actin filaments is closely related to EMT [69]. Actin exists in two forms: a monomeric unit G-actin (globular actin) and a polymeric filament, F-actin (fibrous actin). G-actin is evenly distributed between the cytoplasm and nucleus. G-actin readily polymerizes under certain physiological conditions to form F-actin with the concomitant hydrolysis of ATP. Furthermore, the distribution of F-actin filaments depends on the cell type as well as the cell cycle phases. Cell spreading and adhesion to ECM are mediated by actomyosin by forming prominent bundles of F-actin, which are known as stress fibers. Stress fibers connect to focal adhesions and hence play an important role in cell adhesion and morphogenesis. Actin filaments interact with actin-binding proteins and myosin II within the leading cell edge and deliver F-actin. This provides an important mechanism for cell movement. In the assembly and disassembly of the actin cytoskeleton, myosin II is considered to play a central role through its ATP- dependent motor function [70]. Actin organization is vital for different cellular processes like cell motility, organelle movement, maintenance of cell junctions, and cell shape [71,72].

The EMT process is regulated by gene expression, post-translational modification of proteins, and reorganization of the cytoskeleton [73]. Epithelial cells are held together by tight junctions (TJs), adherens junctions (AJs), and desmosomes and are also connected to the extracellular matrix (ECM) through integrins [9]. Maciej et al. show that endothelial cells maintain cell-cell junctions (adherens and tight junctions) through the stabilization of F-actin. F-actin filaments are stabilized by amplifying β-catenin and the ZO-1 proteins in the cells that overexpress tropomyosin1 [74]. They also show that α-catenin has a role in suppressing actin polymerization in the area of cell-cell junction [75]. Microscopy studies have shown that during early EMT, dynamic changes happen at the cell-cell boundaries, which weaken the AJ and cell-cell adhesion [76], resulting in the destabilization of the cell-cell junctions. Separation starts with the sequential loss of TJ, AJ, and desmosome integrity, commencing the transition of epithelial cells into a mesenchymal state [26]. This transition occurs through variable intermediate-hybrid states [77]. Recent work has revealed that cells in intermediate states of EMT acquire an augmented capacity for tumor-cell dissemination. Molecular markers have characterized these states, but the structural features and the cellular mechanisms underlying these invasive characteristics are yet to be researched.

E-cadherin-mediated cell-cell adhesion complexes are attached to the actin cytoskeleton via β-catenin and α-catenin. E-cadherin complex is destabilized by post-translation modifications, such as increased phosphorylation, internalization and degradation of E-cadherin, and β-catenin [78]. This triggers the destabilization and degradation of AJs. It has also been shown that E-cadherin complexes are attached to the dynamic actin framework via α-catenin and stabilized by suppressing the activity of Rho A and activating Rac and cdc42 [79,80]. On the other hand, cell-surface receptors, such as the integrins, bind to ECM components and play a key role in modifying the cell attachment required for motility and invasion. A multi-protein complex achieves integrin-mediated cell-matrix adhesion and links to the actin cytoskeleton. The majority of integrins, a cell-surface receptor, connect with the actin cytoskeleton in cell-matrix adhesions via cytoskeletal linker proteins, e.g., talin, paxillin, and vinculin [81,82]. FAK is a tyrosine kinase, and it has a role in actin remodeling dynamics during cell adhesion, and motility and its expression and activity correlate with increased metastatic phenotype [83]. These connections between integrins and the actin cytoskeleton are necessary for the activation of downstream pathways. Snail (transcription factor) induces the expression of αvβ3- integrin, which is localized in the invading front of cancer and enhances cell detachment [84]. It has been shown during the EMT process, engagement of integrins by collagen type I results in a loss of E-cadherin mediated cell-cell contact and activation of the β-catenin pathway in pancreatic cells [85]. Thus, integrins provide a link between the outside environment and cellular responses related to motility, such as immune cell trafficking, hemostasis, and migration of cancer cells.

#### 3.1.1. Actin-Binding Proteins

The actin cytoskeleton is a collection of microfilaments (actin) and a vast array of actin-binding proteins (ABPs). Research studies have shown that a major proportion of ABPs travels between the cytoplasm and the nucleus. The ABPs control the process of assembly and disassembly of actin microfilaments. This plays a pivotal role in cell movement, division, membrane organization, and cancer progression, all of which require the coordinated turnover and remodeling of the actin filaments [18]. The polymerization is associated with the formation of flat, sheet-like membrane protrusions called lamellipodia, or finger-like extensions at the edge of lamellipodia called filopodia [19]. In lamellipodia, actin filaments are arranged in a loosely organized meshwork, often referred to as dendritic networks [20], whereas, in filopodia, actin filaments arrange into parallel bundles [21]. These two different types of organizations rely on the action of specific actin-organizing proteins. During migration, actin depolymerization and debranching occur that allow the dynamic remodeling of the actin network and the cyclic extension and retraction of lamellipodia—this generates the pushing force that provides the propulsion for the cell to move forward. Due to the actin filaments’ contraction, the cell body follows the direction of the front lamellipodia. Filopodia are formed of tightly bundled parallel actin filaments with their tapered ends facing toward the plasma membrane. Filopodia filaments are primarily bundled by small crosslinking actin-binding proteins like fascin [22].

The extravasation step is also dependent on actin cytoskeleton dynamics. The entire blood vessel escape process involves attaching the cell to the endothelium, crossing the adhesive endothelium, and then finally establishing a secondary tumor site [33]. It is believed that cells can breach the tissue barrier due to the formation of F-actin protrusions, called invadopodia, that degrade the ECM, thereby enabling cell penetration [34]. We can conclude that lamellipodia and filopodia are involved in the process of forwarding movement of the cell. In contrast, invadopodia are actin-rich protrusions that are associated with the degradation of the ECM through the local deposition of proteases and are involved in cell penetration.

Extensive research has broadened our view on how ABPs affect the rate and extent of polymerization through their wide range of functions—maintaining the pool of monomeric actin (profilin), regulating the state of polymerization of actin filaments (ADF/cofilin), regulating actin filament dynamics and capping (gelsolin, villin), severing actin filaments (cofilin, gelsolin), actin filament nucleation (Arp2/3 complex, WASP), binding to the sides of actin filaments (gelsolin, Arp2/3) bundling and crosslinking (fascin, fimbrin) [86], they have been at the forefront of cancer research. Actin polymerization is a tightly regulated activity. The Arp2/3 (actin-related protein2/3) complex is a seven-subunit protein, controlled by its link with the WAVE and WASP family of WH2 domain-containing proteins (WAVE1, 2, & 3, WASP and N-WASP) that bind both the Arp2/3 complex and actin monomers [87,88]. This, in turn, brings the actin monomers close to the Arp2/3 complex, thereby increasing the rate of Arp2/3-mediated actin polymerization. Arp2/3 is a protein complex involved in the origination of actin filament polymerization. Arp2/3 is frequently overexpressed in malignant tumors, such as breast and liver carcinomas, suggesting a strong correlation between dynamic actin reorganization and cancer progression [89]. WAVE3 is essential for the EMT process to start through the involvement of DNA synthesis, the migration, and the formation of protrusions in breast cancer cells. In-vivo studies show that knockdown of WAVE3 decreases the number of lung metastasis of breast cancer in SCID mice [90,91,92]. Through their binding partner, WASP family proteins, poly-proline motif, also bind profilin, which further helps recruit actin monomers to the Arp2/3 complex. WASH protein is overexpressed in a breast cancer cell line SKBr3 and may be a potential biomarker for EMT [93]. The actin-binding protein cortactin also binds to Arp2/3, and this helps to locate active Arp2/3 complexes to the sides of existing actin filaments leading to branched arrays of F-actin. The overexpression of cortactin has been identified during metastasis [94,95].

ADF/cofilin is often referred to as a depolymerization factor because it is binding to slow-growing ends of actin filaments that accelerate depolymerization. During the EMT process, filopodia are stabilized through the LIMK/cofilin signaling pathway, suppressing actin fibers’ cleavage [69]. Another actin-binding protein fascin is upregulated during migration by stimulating the formation of invadopodia and filopodia [96]. It is reported that gelsolin is required to form lamellipodia and podosomes, which are important protrusions for motile cells [97]. The expression and secretion of urokinase plasminogen activator (uPA), an important protein triggering a cascade to degrade extracellular matrix, is dependent on gelsolin, suggesting the role of gelsolin to enhance invasion [98]. Formins are the actin nucleating proteins that regulate cell movement and organization. It has been reported that formin expression is upregulated at the leading edge in mesenchymal-transformed cells upon EMT [99]. Cortactin, MENA, and Tks proteins form the core structure of invadopodia and play an essential role in actin polymerization, cell signaling, membrane penetration, ECM adhesion, and degradation. Research has also shown that Tks protein is primarily required in invadopodia formation and invasion activity in various human cancer cells [100]. Contactin induces EMT in different types of cancer [101,102]. Karamanou et al. showed that contactin-mediated cell movement induces EMT and participates in tumor migration and invasion [103].

Studies have shown that the gene coding for ABPs displays altered transcription or translation in different cancer types. Alterations in the actin cytoskeleton are a general feature of tumor cells since the ABP expressions are changed in various cancer types. For example, expression of gelsolin, cofilin, CapZ, and thymosin β10 are altered in ovarian cancer, whereas CapZ, CapG, profilin1, cortactin, gelsolin, N-WASp, and WAVE expression levels are changed in prostate cancer [104]. Similarly, data has shown that increased levels of Tβ4 are associated with chronic liver disease and are involved in liver fibrosis by regulating the proliferation and activation of HSC [105]. Other studies have highlighted that increased levels of N-cadherin and decreased levels of E-cadherin have a direct correlation with poor prognosis and cancer progression in prostate cancer patients [106]. Androgen receptor (AR) is associated with EMT and metastasis. In metastatic castration-sensitive prostate cancer, AR deprivation therapy is used as part of combination therapies [107]. Expression of ARPC2 (actin-related protein2/3 complex) is higher in breast cancer tissues, and higher expression of ARPC2 significantly contributes to EMT and metastasis [108]. Research suggests that EMT promotes actin remodeling, which in turn makes the breast cancer cells resistant to NK-cell–mediated killing—actin polymerization is impaired by knocking down N-WASP or CDC42 [109]. Filamin deficit is predominant among carcinomas, including colon, prostate, and breast cancer [110]. Studies have shown that FLNα acts as a promoter in metastasis and invasion in the cytoplasm but acts as a tumor suppressor in the nucleus. Both colorectal and breast cancer cells expressed a high level of TAGLN (22-kDa actin-binding protein). This, in turn, enhances migration and correlates with poor prognosis [111,112]. Higher expression of α-actinin (actin filament cross-linker) is associated with poor prognosis in breast cancer and significantly associated with the degree of clinical advancement and lymph node status [113,114].

#### 3.1.2. Rho GTPases

During EMT, actin and MT dynamics are regulated by Rho GTPases. In different types of cancer, it has been shown that high expression of Rho GTPases genes correlates with a metastatic phenotype. The intracellular protein signaling cascades control the binding of the monomeric or polymeric form by actin-binding proteins (ABPs) when the GTPases of the Rho family are activated. The Rho family of GTPases, including Rac1, RhoA, and Cdc42, are well known for their regulation of actin cytoskeleton organization— such as cytoskeletal dynamics, directional sensing, cell-cell junction assembly/disassembly, and integrin-matrix adhesion. Controlling the activities of Rho GTPases is critical during the growth-factor-induced EMT. Rho activates actin stress fiber formation and regulates cytoskeleton changes, affecting cell-cell or cell-matrix adhesion. The role of Rho signaling in the regulation of actin-myosin contraction is vital and causes actin reorganization to induce stress fibers. Rho-associated kinase (ROCK) promotes myosin light chain phosphorylation, activates actin severing factor cofilin through LIM kinase. Rho signaling pathway affects the activity of various other proteins that control actin polymerization, including profilin and FH proteins [115]. Rac1 stimulates lamellipodia formation through regulating branched actin polymerization [116]. Both Rac1 and Cdc42 are present in the front edge towards the direction of migration. Cdc42 is also upregulated in different types of cancer [117,118]. Hepatocyte growth factor (HGF) activates CdC42 and Rac, which induces filopodia and lamellipodia formation and cell scattering through actin cytoskeleton rearrangement [119]. TGF-β induces activation of RhoA, results in disruption of cell-cell adhesion and formation of stress fibers [120]. RhoA is also involved in microtubules-mediated cell-matrix adhesion and basement membrane integrity [121]—multiple downstream effectors of Rac, Cdc42, and RhoA participating in the reorganization of the actin cytoskeleton. Recent studies have shown that many of these regulatory pathways become deregulated in cancer cells and most likely add to the invasive behavior during metastasis [122].

### 3.2. Microtubule (MT)

In EMT, the aspects of the actin cytoskeleton and intermediate filaments are well identified, but the functions of microtubules (MT) are still under discovery. MTs are significant parts of the cytoskeleton, play an essential role in motility, intracellular trafficking, and support the cell shape [123]. MTs are composed of α and β-tubulin dimers, and they mostly grow and shrink from the plus end and create dynamic instability [124]. The functions of MTs are dependent on their assembly and stability, which are regulated by post-translation modification and interaction with various stabilizing and destabilizing proteins [125].

The stability of the MT network has been involved in the control of reattachment and cell migration through α-tubulin acetylation at lysine40 (post-translation modification). The acetylation leads to the formation of cell protrusion and tumor cell reattachment, which promote breast cancer metastasis [65]. Calmodulin-regulated spectrin-associated protein (CAMSAP3) is an MT binding protein required to maintain MT organization. It has been shown that loss of CAMSAP3 promotes Akt-dependent EMT by tubulin acetylation [126]. In the EMT program, the transcription factors (TWIST and SNAIL) expressions are enhanced, promoting α-tubulin detyrosination. This further promotes the formation of tubulin-based micro tentacles. These then enhance the reattachment of circulating tumor cells to the endothelial cells [127].

Research has shown that microtubule-interacting protein EB1(end binding protein) co-localizes and interacts with microtubules. EB1 is a negative regulator of microtubule stability and promotes tumor cell migration. It modulates MT dynamics both in vitro and in vivo [128,129,130]. Furthermore, MT-associated protein ATIP3 is encoded by the tumor suppressor gene MTUS1. Breast cancer cell migration is enhanced by loss of ATIP3 and associated with altered MT dynamics [131]. Decreased expression of ATIP3 inhibits MT tips from reaching the cell cortex during migration, which is essential for cell polarity and migration [132]. ATIP3 is used as an important prognostic marker for breast cancer patients.

Stathmin is an MT regulator protein that depolymerizes MT and enhances and regulates MT dynamics. The destabilization of MT is linked to the phosphorylation of stathmin at its four serine residues [133]. In some human cancer, like sarcomas and Wilms tumors, stathmin has been upregulated and linked to more aggressive metastasis [134].

During EMT, MT has a significant role in cell migration. The anti-MT drugs work via inhibiting cell division on the one hand but also by inhibiting cell migration by stopping the forming of MT network-based membrane protrusions [135,136]. Several strands of research exist on the role of drugs in cell migration [137,138]. It has been shown that the subtoxic dosage of drugs reduces only cell migration without affecting cell division. However, a higher concentration of drugs inhibits cell division but exhibits loss of directionality. It has been shown that MT restrains cell movement as well as establishes directionality [139,140]. But despite the various bodies of research as stated before, the complete role of MT in EMT is still not fully understood, and further research needs to be done to analyze how MT dynamics are correlated with EMT.

During cell migration, one of the critical phenomena is the interaction of MT with the actin cytoskeleton [141]. In response to extrinsic signals, cells migrate due to the activity of RhoA, Rac1, and Cdc42 and their downstream targets. This further mediates a change in the actin cytoskeleton and maintains the stability of MT. Furthermore, variation in MT stability regulates cortical F-actin through activation or inhibition of different Rho GTPases [142].

Apart from their role in cell division and migration, MT also plays an essential role in cell polarization. Research studies have shown that MI s are nucleated at their minus ends, which confines mostly at the centrosome, and the plus ends are stabilized at the leading edge. It had been shown that selective stabilization of the plus ends of MTs enables the centrosome to reorient towards one particular leading edge, which results in a directed movement of the cell [132,143]. Cortical regulation of MT supposedly plays a vital role in creating a polarized MT needed for morphogenesis and cell migration. MT indirectly contributes to cell-cell adhesion by dynamic remodeling of actin network, but the role of MT to function with cell-cell adhesion to regulate migration or EMT is still under active research. Byrne et al. show that MT-interacting protein stathmin is involved in cell migration and metastasis through crosstalk between MT and actin cytoskeleton [144]. Utilizing this interaction where the actin cytoskeleton is targeted via MT, novel pharmacological strategies could be designed that could surpass the toxic effects associated with some actin-based therapies.

### 3.3. Intermediate Filament (IF)

Intermediate filaments (IFs) are essential cytoskeleton components that give structural support and mechanical strength. More than 50 different IF proteins are encoded by one of the largest families of genes in the human genome, which inscribe five different categories of IF. Types I–IV are localized in the cytoplasm, which includes vimentin, which is a classical marker for EMT, and its expression correlates with the invasive phenotype of epithelial cancers. In order to maintain cell shapes, IFs are associated with the plasma membrane and other elements of the cytoskeleton [145]. IFs also exhibit distinct tissue expression patterns as compared to the actin cytoskeleton and MT.

Type I IF keratins are epithelial-specific and essential for the mechanical stability of epithelial cells. These filaments are associated with desmosome and hemidesmosome through a complex network that extends from the periphery of the nucleus to the plasma membrane. During EMT, the reduction of keratin proteins is often considered a histological and biochemical feature of cancer cells [146,147]. Desmosomes are essential for epithelial integrity, and keratin stabilizes desmosome-mediated intercellular contacts [148]. In epithelial cells, the expression of vimentin activates the destabilization of desmosomes and increases focal adhesion dynamics to promote migration [149].

A type III IF, vimentin, is a canonical marker of the EMT. Vimentin expression is upregulated during EMT in epithelial cells, and increased vimentin expression has been reported in various cancer cell lines and tissues, including prostate cancer, breast cancer, malignant melanoma, and colorectal cancer. It is used as an indicator of poor prognosis [71,150,151]. In the reverse process, the mesenchymal to epithelial transition (MET), vimentin expression is downregulated as cell motility decreases, and cells get epithelial morphology [152]. During EMT, vimentin contributes to the determination and maintenance of cell shape. In breast cancer, vimentin plays a significant role in the EMT processes, and its knockdown results in a reduction in genes linked with breast cancer invasion and the basal-like phenotype [153]. Recent studies have revealed that vimentin expression is linked with motile prostate cancer cell lines, and its knockdown significantly decreases tumor cell motility and invasive activity [154]. Xuan et al. show that vimentin expression is significantly high in polyploidal giant cancer cells (PGCCs). Vimentin intermediate filament is responsible for enlarged morphology and increased migration [155]. Collectively, vimentin expression is preeminently characterized in the EMT process, including tumor cell migration and invasion.

A type VI IF, nestin, is known as a stem cell marker in embryonic and adult central nervous system (CNS) stem cells [156]. Furthermore, from recent research, the role of nestin has been amplified to show that it is also a CSC marker in different forms of cancer, like brain tumors, ovarian, glioblastoma, lung tumors, and head and neck cancers [157]. It has been shown that nestin interacts with IFs like vimentin and desmin to form heterodimers or polymers (which provide cellular support, maintain cellular membranes), and regulate apoptosis-related factors that support cytoskeleton reorganization during mitosis [158]. It has been shown that nestin is involved in the cellular migration and metastasis processes by modulating E-cadherin and Snail expression [159]. Nestin is essential for TGF-β1/Smad mediated EMT in pancreatic cancer. Overexpression of nestin is a positive feedback regulator of the TGF-β1 signaling pathway. This implies a significant role of nestin in the regulation of TGF- Induced EMT, thereby serving as a potential treatment for pancreatic cancers [160,161].

Furthermore, data from clinical samples (shown in Table 1) establishes the cytoskeleton’s role in the EMT process [162]. Table 1 shows the correlation between cytoskeleton genes and EMT of various cancers in clinical samples. The data shows about 65% of cytoskeleton genes are positively correlated with EMT.

## 4. Clinical Evidence for the Actin Cytoskeleton in EMT and Therapeutic Implications

There are primarily two forms of drug resistance in cancer: intrinsic and developed resistance. Intrinsic, as the name suggests, exists before the start of any cancer therapy, and it results in the ability of cancer cells to survive any drug treatment [163,164]. Developed resistance, on the other hand, is when the patient shows an initial positive response to treatment, but, over a period of time, the cancer cell acquires protein alterations, which results in unresponsiveness to treatment [165,166]. Recent studies have shown that scientists focus on combination therapy targeting multiple molecules in the same signaling pathway, multiple pathways in the same tumor, or targeting both cancer cells and immune cells [167,168]. Combination therapies are still under investigation and will eventually better our understanding of drug resistance mechanisms. Different pharmacological strategies have been used to target EMT, such as extracellular inducers and transcription factors. Both have some advantages but also have some distinct drawbacks. For instance, direct inhibition of transcription factors (TF) has been chemically challenging, and successful studies regarding direct targeting of EMT -TFs have been few and far between. The efficacy is limited due to the presence of a large variety of TFs that can initiate EMT. The other disadvantage is that this needs to be initiated in the early stages of carcinoma [169,170]. Hence, new ideas have suggested that targeting EMT and cytoskeletal proteins would be novel in combating cancer drug resistance.

Actin is essential for normal cell physiology. Hence potential actin-specific chemotherapies, despite their promise in-vitro and in-vivo, have not been successful due to their nonspecific targeting of normal tissues causing cardiotoxicity and renal problems [171,172]. Recently it has been shown that anti-tropomyosin compounds, which only target tropomyosin-containing filaments in cancer cells, can be used to treat a wide variety of cancer [173]. Studies have revealed that suppressing ROCK, LIMK, and cofilin inhibit cancer metastasis. Over the years, inhibitors of ROCK, LIMK, and cofilin have been investigated in preclinical and clinical models as anti-cancer agents. A few inhibitors, such as Y-276432, have been developed for ROCK1/ROCK2 and prevent MDA-MB-231 breast cancer cell metastasis. Fasudil is the only clinically approved ROCK inhibitor used in humans for systemic applications [174]. MRCK regulates actin-myosin contractility and has a role in cell invasion and metastasis. BDP5290, a potent inhibitor, strongly inhibits the invasion of human squamous cell carcinoma [175].

Similarly, JG-6, an oligosaccharide, is a cofilin-inhibitor that can induce actin depolymerization and suppression of migration and metastasis in MDA-MB-435 and an orthotopic xenograft model [176]. Increasing evidence suggests that the increase in the level of EMT-related actin-binding proteins (ABPs) associated with the actin cytoskeleton reorganization is due to the initiation of the EMT process and metastasis. Therefore, management of ABP expression can possibly help in suppressing migration and promote cancer cells’ sensitivity towards drug treatments. Many studies have focused on Arp2/3, cortactin, formins, and fascin. However, the role of other ABPs, which can also be potential targets in carcinogenesis, is understudied. Another challenge for anti-cancer therapy is that the actin cytoskeleton and ABPs are difficult to target actin, and ABPs are involved in the formation of contractile structures in cardiac and skeletal muscles [104].

Intermediate filaments vimentin and nestin are associated with different types of cancer. Vimentin is a marker for mesenchymal cells while participating in EMT. Withaferin-A, a naturally derived anti-cancer drug, works by apoptosis induction in vimentin expressing cancer cells [177,178]. Another drug, moscatilin, is proven to inhibit EMT and sensitizes anoikis, causing programmed cell death [179,180]. Another recently discovered drug, FOXC3 inhibiting vimentin effector1(FiVe1), shows promising results, and this specifically targets vimentin [181].

Anti-tumor drugs are found to alter microtubule dynamics, which subsequently affect mitosis and apoptosis [182]. Taxol was known to be the first drug to initiate tubulin assembly and inhibit microtubules disassembly from halting mitosis [183]. Nanoparticle albumin-bound paclitaxel (Abraxane^®®^) is an intravenously administered microtubule inhibitor. Nab-paclitaxel plus gemcitabine have been shown to have a good outcome in metastatic pancreatic cancer. This combination of drugs leads to a break in the cells’ reproduction activity [184]. In children with recurrent neuroblastoma, ABT-751, a type of orally active drug, works by inhibiting microtubule polymerization by binding to β-tubulin [185]. During EMT, microtubules have significant control in tumor migration and invasion. These anti-tumor drugs inhibit cell division and formation of the membrane protrusions formed by the network-based microtubules, which trigger cell migration and invasion. Eribulin is a MI depolymerization drug used to treat patients with metastatic breast cancer. This drug inhibits angiogenesis, vascular remodeling, and EMT in breast cancer [186,187]. The compound 2-hydroxy-4-methoxy-2′,3′-benzochalcone (HymnPro) disrupts microtubule assembly, which leads to mitotic arrest and progressive activation of the caspase pathway leads to the anti-tumor property, ensuing apoptosis [188]. BPR0C305 is an orally active drug that inhibits tubulin polymerization and disrupts cellular microtubule assembly [136]. The diaryloxazole PC-046 is an anti-tumor drug also with high oral bioavailability. It is a small molecule microtubule destabilizing agent that is synthetically derived. This drug is known to have the advantage of having fewer MDR cross-resistance compared to other prevailing microtubule destabilizing agents [135]. Table 2 summarizes the various drugs that target the cytoskeleton protein, including their specific mode of action.

### EMT Related Cytoskeleton Proteins- Associated Multi-Drug Resistance (MDR)

Multiple signaling pathways involved in EMT and cytoskeletal proteins play an important role in drug resistance in cancer cells [199]. EMT cells have an increase in anti-apoptotic effects and drug efflux pumps. Hence, new ideas have suggested that targeting EMT and cytoskeletal proteins would be novel in combating cancer drug resistance. Chemotherapy is widely used in cancer treatment as monotherapy or as a combination with radiotherapy or surgical intervention. In recent years, multiple discoveries have been made in cancer treatment as drug resistance, which has been one of the significant causes of cancer mortality, is on the rise [200,201,202]. Many targeted therapy drugs (e.g., Erlotinib, Gefitinib) have shown promising results during the initial trials. However, the majority of them develop drug resistance after long-term drug therapy [203].

EMT and drug resistance have been associated with one another for almost two decades [200]. Multiple findings have shown a significant link between metastatic cancer cells and EMT. One of the common reasons that have been a significant impediment to the success of cancer pharmacotherapies is the overexpression of ATP-binding cassette (ABC) efflux transporters in cancer cells. Recent studies have established that angiopoietin-like 4 (ANGPTL4) protein plays a pivotal role in the metastatic distribution of cancer cells and boosts MDR in the cancerous cell during the EMT process by transcriptionally upregulating the ABC transporters expression via the Myc and NF-κB signaling pathways [204]. It has also been observed that ANGPTL4 increases ABC transporter activity, which results in pushing anticancer drugs out of cells, which causes chemotherapy failure. However, the function of ABC transporters beyond their drug-efflux capacity remains mostly unexplored. There are various schools of thought, and one of the ways suggested to overcome MDR is to develop ABC efflux transporter inhibitors to alert cancer cells to chemotherapeutic drugs [55].

EMT cells are speculated to have selective growth ability in the drug-filled environment. Though some papers suggest that EMT does not entirely contribute to cancer metastasis, other papers show that drug resistance in cancer cells is highly associated with EMT. This includes bladder cancer [205], pancreatic cancer [7], breast cancer [206], lung cancer [207] and ovarian cancer [208].

Adriamycin-resistant MCF-7 cell lines and vinblastine-resistant ZR-75-B cell lines are shown to have undergone EMT, whereby adriamycin-resistant MCF-7 cells show high vimentin levels and have suppressed the formation of desmosomes and tight junctions, which are specific phenotypes for EMT. [209]. Recent studies have focused on ACTN4, an actin-binding protein whose expression increases with cell motility and EMT. It has been shown that during EMT, ACTN4 interacts with Akt signaling, and this may lead to resistance to DNA damaging drugs, which are used in cancer therapy [210,211].

**Figure 3:**
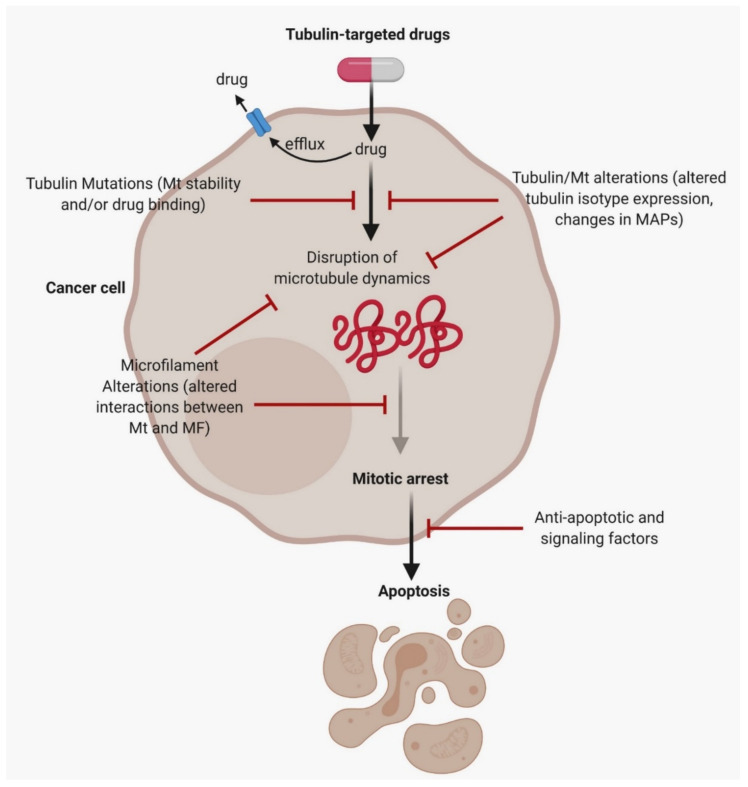
A schematic diagram illustrating the resistance mechanisms associated with anti-microtubule drugs. These drugs cross the lipid bilayer of the cell membrane and then bind themselves to β-tubulin to alter the microtubule dynamics, causing mitotic arrest and consequent apoptosis. Drugs can be effluxed before reaching the cellular target with the aid of functional drug transport protein. Disruption in the tubulin/microtubule system can avert anti-microtubule drugs from disrupting the microtubules and leading to drug resistance. Before drug binding occurs, signaling and anti-apoptotic factors may also contribute to drug resistance. MF: microfilaments, Mt: microtubules. [212]. The figure was created with BioRender.com (Accessed on 1 February 2021) and was exported under a paid subscription.

A vast body of research has described that modifications in the drug target, such as changed microtubule dynamics, tubulin mutations, modified tubulin isotype expression, and altered microtubule regulatory proteins, are the critical targets of anti-microtubule drug resistance. Research has also indicated that other cytoskeletal proteins that can regulate microtubule regulations through signaling or structural connections may be essential factors of anti-microtubule resistance [212]. This resistance to anti-microtubule agents can be either congenital or acquired over the years due to the mentioned factors. The following (Figure 3) is a schematic diagram that illustrates the resistance mechanisms associated with anti-microtubule drugs.

**Figure 4:**
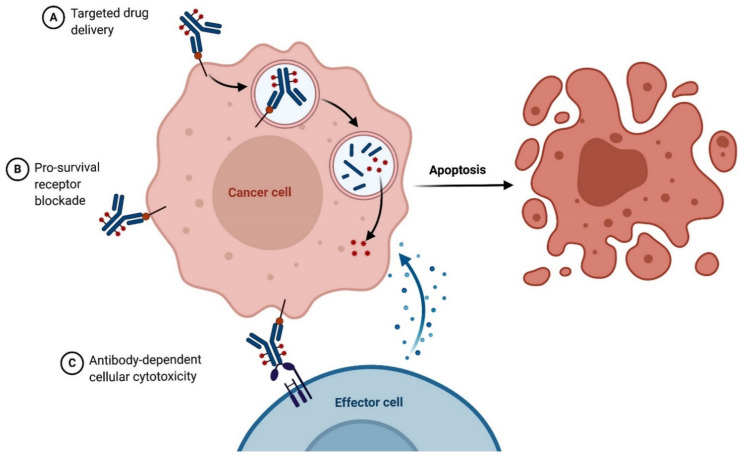
A diagram illustrating the mechanism of action of Antibody-Drug Conjugate (ADC) in a cancer cell. (**A**): High-affinity Antibody binds to the drug, forming ADC, entering the double lipid-membrane layer of the cell to cause cell death. (**B**): ADC binds to the pro-survival receptor of cancer cells, inhibiting its function, commencing apoptosis. (**C**): ADC binds to both the membrane-surface antigen of the cancer cell and an effector cell in the immune system, inducing cellular cytotoxicity lysing the cancer cells. The figure was created with BioRender.com (Accessed on 1 February 2021) and was exported under a paid subscription.

Antibody-drug conjugates (ADCs) are a new class of targeted anticancer therapy found to be efficient in MDR cancer. A key mode in which these ADCs cause apoptosis in tumor cells is when high-affinity antibody (Ab) couples with the drug and drives a targeted drug delivery into the cell. This Ab-drug conjugate also blocks the cells’ pro-survival receptor besides forming a cytotoxic load coupled by a selective tumor cell killing (Figure 4). Initially, two ADCs—Mylotarg and Adcetris—were approved by the US FDA for treating hematological malignancies. However, the significant discovery was when breast cancer-targeting ADC, Kadcyla was found. To improve the efficacy and attenuate the side effects, integrated ‘drug: antibody ratio’ (DAR) has been attained [213]. In a discovery by Endo et al. on ADC, cytoskeleton-associated protein 5 (CKAP5), which is a microtubule-associated protein, has been shown to serve as a cell surface target for T-DM1. The binding of these two molecules is mediated by payload (DM1). Upon forming this complex, cell membrane damage occurs which leads to calcium influx, disrupting microtubule network, and apoptosis [214]. The discovery of ADC can lead to other combination therapies, including immunotherapy. Extensive research is currently ongoing to develop strategies to enhance the efficacy and targetability of ADCs in treating tumors. We conclude this section with a table (Table 3) that shows anti-cancer drugs which target the cytoskeletal proteins to alter or inhibit EMT in cancer therapy.

## 5. Conclusions

EMT is a highly active process of conversion of epithelial cells to mesenchymal cells. The transformation in the phenotype of an epithelial cell to mesenchymal involves the cell gaining features such as invasiveness, motility, multi-drug resistance, immune-evasiveness, and immunosuppressive properties. In turn, the cell migration occurs because of the swift reorganization of the actin cytoskeleton consisting of polymerization and disintegration of actin filaments. Research findings suggest the vital role of actin-binding proteins in regulating the polymerization and depolymerization process of actin filaments [86]. Studies have also proved that the loss of E-cadherin, which localizes the adherens junctions, is one of the critical features in EMT transitions. The Rho family GTPases also play a vital role in controlling the dynamics of the actin cytoskeleton in both epithelial and mesenchymal cells. Numerous studies have also shown that actin-binding proteins (ABPs) perform many distinct functions that affect the rate and extent of polymerization—nucleating, capping, severing, sequestering, bundling, and crosslinking [86]. Over the years, studies have elucidated that microtubules and intermediate filaments also play a vital role in EMT. Microtubules play an essential role in motility, intracellular trafficking, supporting the cell shape, and produce pushing and pulling forces to support protrusion. The role of intermediate filaments has been a subject that has evoked much interest in recent years. There is clear evidence that points to the fact that EMT is associated with vimentin protein expression, which undergoes phosphorylation and reorientation in cells, regulating cell contraction and focal adhesion assembly/disassembly.

Furthermore, crosstalk between different components of the cytoskeleton is present during metastasis. Actin, IF, and MT cytoskeletons work together in cell migration and metastasis [234]. Recent evidence paints a strong relationship between cytoskeleton dynamics and EMT, which can be utilized to identify potential biomarkers.

## 6. Future Perspectives

In recent years, it is gradually becoming evident that targeting EMT in cancer treatment may lead to new targets for the development of anti-cancer therapies. In recent research, it has also emerged that several metastatic and invasive cancer have lacked signs of EMT [235] (loss of epithelial feature or increase of mesenchymal proteins). Hence, further research must be done to understand the mechanism of the underlying regulation of actin cytoskeleton and cancer cell EMT. For all these reasons, actin presents itself as a hypothetically attractive anti-cancer therapeutic target. However, in reality, results have proved that actin has been a poor target because of toxic side effects primarily due to the inability of therapeutics to distinguish between actin isoforms [236]. In the past few years, pharmaceutical research studies have pivoted their direction from actin to actin-binding proteins such as the Arp2/3 complex and tropomyosin, which are promising therapeutic targets in cancer drug discovery plans, as these proteins offer many isoforms for selective targeting and the prospect to avoid toxic side effects [237]. Recent studies have highlighted a key characteristic of the protein that makes it a lucrative candidate for further research as a therapeutic target, its specific modulation in activity levels and expression in cancer cell lines. Examining actin-binding proteins as novel therapeutic targets offer great potential for the development of specific cancer therapies—researchers also need to consider a lot of procedural considerations when using phenotype screening to obtain positive outcomes. These new findings and analysis are an active area of interest since it can lead to breakthrough results—combining conventional cancer therapy with EMT-related mechanisms in our fight against cancer and drug-resistant cancer cells.

## Figures and Tables

**Table 1:** Correlation (Rho) of cytoskeleton genes expression (FPKM) with EMT score in clinical samples (TCGA cohorts, *n* = 12,290.) A higher Rho indicates that a sample has more mesenchymal-like phenotype, whereas a lower Rho indicates that a sample has a more epithelial-like phenotype. The correlation significance was assessed using the Spearman correlation coefficient test.

Gene	Spearman Correlation Coefficient Rho	Spearman Correlation Coefficient *p*-Value
ACTB	−0.044	8.75E−07
ACTR2	−0.124	5.61E−44
GSN	+0.220	6.81E−135
LIMK1	+0.168	1.28E−78
CFL1	−0.207	3.13E−119
WASF1	+0.509	0.00E+00
FN1	+0.152	5.68E−65
CTTN	−0.392	0.00E+00
WAS	+0.413	0.00E+00
WASL	−0.027	2.11E−03
FSCN1	+0.329	0.00E+00
TUBA1A	+0.656	0.00E+00
VIM	+0.593	0.00E+00
NES	+0.580	0.00E+00
TPM1	−0.129	3.78E−47

Gene expression (FPKM) data from TCGA cohorts were downloaded from Broad firehose, version 2016_01_28 (Reference: Broad Institute TCGA Genome Data Analysis Center (2016): Firehose stddata__2016_01_28 run. Broad Institute of MIT and Harvard. doi:10.7908/C11G0KM9). The EMT score was computed using a previously defined EMT signature and the two-sample Kolmogorov-Smirnov-based method [162].

**Table 2:** Examples of various drugs targeting cytoskeletal molecules and their action mechanism.

Cytoskeleton Target Protein		Drug Therapy	Mode of Action	References
Actin		Cytochalasins	Inhibits polymerization by binding F-actin	[189,190]
		Latrunculin	Inhibits polymerization Enhances depolymerization through interaction with G-actin.	[191]
		Jasplakinolide	Enhances polymerization by binding F-actin at multiple sites.	[192,193]
Actin-binding Protein	Tropomyosin	TR-100	Inhibits tropomyosin (TPM3.1) in the tropomyosin-dependent actin filament function to promote anti-cancer drug development.	[194]
	ROCK1/ ROCK2	Y-276432	Inhibits the kinase activities of ROCK1/ROCK2.	[195]
	ROCK1	Fasudil	Inhibits ROCK in the vascular system and is a calcium channel blocker.	[196]
	Actin-Myosin	BDP5290	Blocks MLC phosphorylation on stress fibers and actin bundles.	[175]
	Cofilin	JG-6	Induces actin depolymerization and suppression of migration.	[176]
	LIMK1	4-Pyridocarbazolone	Inhibits cofilin and actin dynamics.	[197]
	LIMK1 and LIMK2	CRT0105950, CRT0105446	Inhibits cofilin phosphorylation.	[198]
Intermediate Filament		Withaferin-A	Binds and inhibits vimentin.	[177,178]
		Moscatilin	Suppresses AKT phosphorylation and also suppresses the expression of vimentin, SLUG, and SNAIL. Inhibits EMT and sensitizes anoikis.	[179,180]
		FOXC3(FiVe1)	Promotes vimentin disorganization, leading to mitotic catastrophe.	[181]
Microtubules		Taxol	Promotes tubulin assembly and inhibits MT disassembly from halting mitosis.	[184]
		ABT-751	Inhibits MT polymerization by binding to β-tubulin.	[185]
		Eribulin	Inhibits angiogenesis and vascular remodeling and is an MT depolymerization drug.	[187]
		BPR0C305	Inhibits tubulin polymerization and disrupts cellular microtubule assembly.	[136]

**Table 3:** Anti-cancer drugs which target the cytoskeletal proteins to alter or inhibit EMT in cancer therapy. The associated cancers and drugs which are resistant to these cancers are also laid out.

Target Cytoskeletal Proteins	Features	Functions	Anti-Cancer Drugs	Function of Anti-Cancer Drugs	Associated Cancers	Drugs Resistant to These Cancers
Vimentin	Central intermediate filament (IF) protein of mesenchymal cells	Organizer of several critical proteins involved in attachment, migration, and cell signaling	Moscatilin FiVe1	Inhibits EMT and sensitizes anoikis FiVe1 disrupts mitotic progression	Lung cancer [179,180] Brest cancer [181]	
α-Actinin	Cellular protrusions, stress fibers, lamellipodia, microvilli, invadopodia of multiple cell types	Crosslinks actin into parallel bundles by forming dimers head to tail	Not Available clinically		Expression in breast, ovary, pancreas, lung, astrocytoma cancers. [55,215,216,217,218,219,220]	Docetaxel, carboplatin, tamoxifen (Ovary and breast) [221,222]
γ-actin	Distributed along perinuclear and nearby cytoplasm, suggesting a distribution based on diffusion or restriction to nearby cytoplasm. [223]	Regulates cellular morphologies, extending processes, and ruffling edges that reflect cell movement [223]	Not Available clinically		Acute lymphoblastic leukemia	Vinblastine, Desoxyepothilone [224]
		Distributes β-actin and form actin-rich retraction fibers during mitosis	Paclitaxel	Targets the microtubule and causes mitotic arrest and apoptosis	Breast cancer [225]	
					Neuroblastoma	Paclitaxel, vinblastine, epothilone [224]
F-actin	Formed by the polymerization of G-actin under physiological conditions, with the concomitant hydrolysis of ATP.	Cell adhesion, migration, and division	Jasplakinolide (Jas)	Stimulates actin polymerization but disrupts F-actin fibers	Breast and prostate cancer [226,227]	
Eplin	Stress fibers of multiple cell types	Actin filament bundling and side-binding	Not Available clinically		Downregulation correlates with progression and metastasis in prostate cancer. Potential tumor suppressor in breast cancer [228,229,230]	
β-Tubulin	polymerize into microtubules, a significant component of the eukaryotic cytoskeleton	Involved in many essential cellular processes, including mitosis	Taxanes (paclitaxel), epothilones, and Vinca alkaloids	Binds to β-tubulin and disrupts microtubule dynamics by inducing a potent mitotic block and subsequent cell deathVinca alkaloids inhibit MT polymerization.	Breast, ovarian, lung cancer [212]	
					Acute lymphoblastic leukemia	Vincristine, vinblastine and desoxyepothilone B [231,232]
					Non-small cell lung cancer	Paclitaxel [233]
Cytoskeleton-associated protein 5 (CKAP5)	A microtubule-associated protein which is encoded by the CKAP5 gene	Regulates microtubule organization, nucleation, elongation, and microtubule dynamics by binding to the plus end of the microtubule. Serves as a cell surface target for T-DM1	T-DM1	Upon forming the T-DM1-CKAP5 complex, cell membrane damage occurs, which leads to calcium influx, disrupting microtubule dynamics causing apoptosis	Heptocellular carcinoma [214]	
